# The curious origins of a high-stress training technique mainlining: its molecular, biochemical, and agronomic perspectives for the cultivation of *Cannabis sativa*

**DOI:** 10.1186/s42238-025-00339-y

**Published:** 2025-11-29

**Authors:** Grace N. Ijoma, Thulani Mannie, Weiz Nurmahomed, Pierre Adriaanse, Memory Tekere

**Affiliations:** Department of Environmental Sciences, College of Agricultural and Environmental Sciences, University of South Africa, Roodepoort, FL 1709 South Africa

**Keywords:** Cannabis, Apical dominance, Mainlining, Stress training, Yield optimization

## Abstract

Mainlining is a high-stress training (HST) technique utilized in *Cannabis sativa* cultivation to restructure plant architecture, enhance canopy uniformity, and increase inflorescence yield. Despite its widespread application, scientific literature detailing the possible molecular, physiological, and agronomic mechanisms underlying this method remains limited. This review consolidates current knowledge on mainlining, focusing on its origins and its interaction with apical dominance, shoot apical meristem (SAM) regulation, and vascular differentiation. The technique involves strategic decapitation to disrupt apical dominance, initiating hormonal and metabolic shifts—particularly in auxin and sugar signalling—that stimulate axillary bud outgrowth and promote symmetrical cola development. Mainlining integrates both low- and high-stress training methods, including topping, lollipopping, and tie-downs, to optimize light distribution, canopy structure, and resource allocation. Further emphasis is placed on the role of vascular remodeling and secondary cell wall development in plant recovery and structural reinforcement following stress. The review also identifies critical research gaps, such as the absence of standardized protocols across *Cannabis* subspecies, and outlines future directions involving omics technologies, AI-assisted cultivation, and precision breeding. This synthesis provides a foundational reference for aligning empirical cultivation practices with plant developmental biology, contributing to the advancement of evidence-based *Cannabis* horticulture.

## Introduction

*Cannabis sativa* is an annual dioecious herbaceous plant indigenous to Central Asia with a recorded history of at least 12,000 years. It was disseminated globally by nomadic communities (Crocq 2020). The extensive use of this plant can be attributed to its numerous functions for textile fibre, food, veterinary nutrition, cosmetics, recreational substance owing to its psychoactive characteristics, and medicine (Crini et al. 2020; Fordjour et al. 2023). *Cannabis* spp. offers a wide array of valuable phytochemicals, with terpenes, such as humulene, being the most renowned for their aromatic qualities (Hanuš and Hod 2020), and cannabinoid compounds, known for their psychoactive and therapeutic effects. The cannabinoid profile comprises several chemicals, with Δ−9-tetrahydrocannabinol (THC) and cannabidiol (CBD) being the two primary cannabinoids. These molecules specifically interact with the endocannabinoid system receptors 1 in the brain and receptors 2 in the body (Capodice and Kaplan 2021). The high-value components CBD and THC contribute to the economic significance of the plant.

Various agronomic and physiological strategies have been explored to improve the yield of *Cannabis sativa*, with mixed success. These include efficient irrigation systems (Bajić et al. 2022), temperature regulation (Herppich et al. 2020), and the induction of physiological stress through nutrient limitation and controlled environmental conditions. Additionally, microbial inoculants, plant density optimization, sowing time adjustments, and fertiliser regimes have been investigated (Farnisa et al. 2023; Pandey et al. 2015). Recently, the application of elicitors to trigger secondary metabolite production has gained attention (Trono 2024; Mansouri et al. 2016). A common trend in present times is that *Cannabis* cultivators share suggestions in a variety of informal online blogs on the efficient techniques they use to enhance their crop yields. Among these techniques, two classifications are prominent and have been successfully implemented in *Cannabis* cultivation, namely high stress and low stress training, as illustrated in Table 1. Low stress training (LST) introduces stress with minimum trauma to the plant. They include gently bending the stems and securing them in place, often with ties, to expose more bud sites to light and encourage horizontal growth. Since the plant undergoes minimal stress, recovery time is usually brief, and there is less risk of stunting growth. LST primarily seeks to optimize light exposure and generate a balanced canopy, which can result in higher yields. High stress training (HST) is aggressive and causes long-lasting stress to the plant. HST includes practices such as topping (removing the top of the main stem) and super cropping (deliberately injuring the stem to encourage thicker branch growth). An advanced type of topping is the manifolding (mainlining) technique. In this method, plant topping is done at least three different times over a period to achieve a range of 2–64 colas. Plants need extended recovery time. If HST is done incorrectly, it could cause permanent harm to the plant. Its purpose is to change the plant's morphology and encourage multiple main colas and boost its overall resilience, and it is mandatory to achieve a screen of green (SCROG) (Trancoso et al. 2022; Caplan et al. 2018). 

**Table 1:**
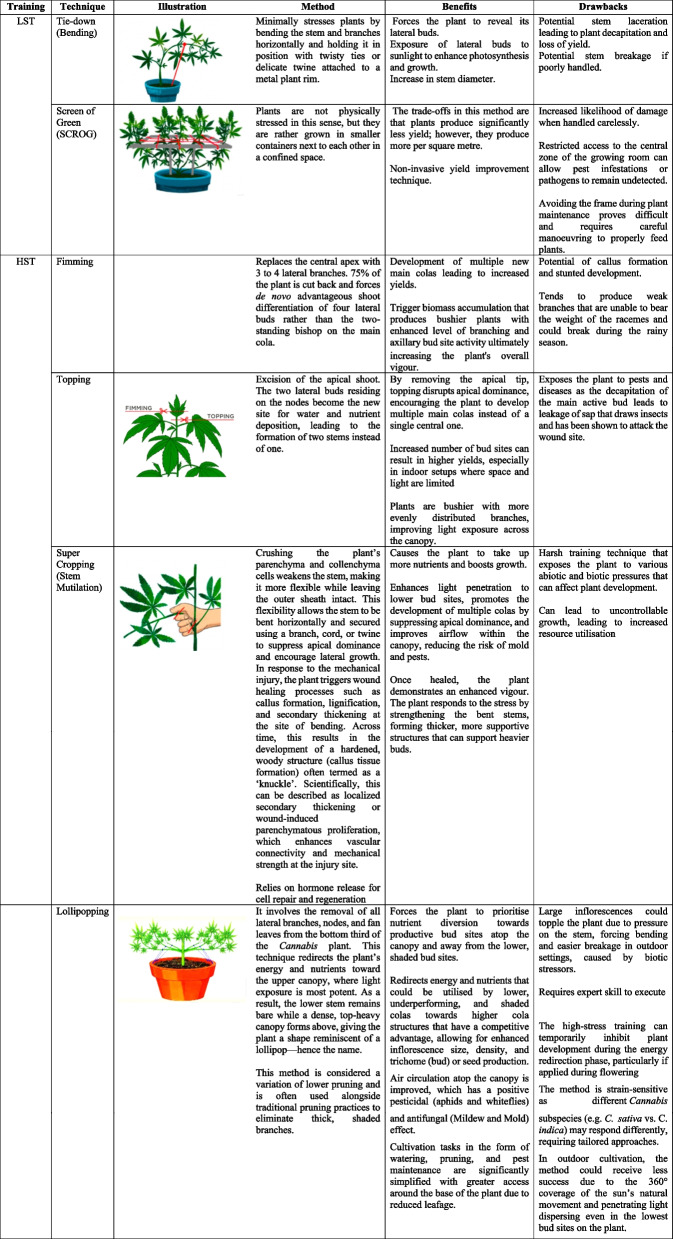
Overview of LST and HST as Components of Mainlining in Cannabis Cultivation (Haze 2024; Beveridge et al. 2023; Trancoso et al. 2022; Danziger and Bernstein 2021; Voser 2020; Spitzer-Rimon et al. 2019)

*Cannabis* cultivation has historically been shrouded in secrecy due to longstanding legal restrictions, and it remains prohibited in several countries worldwide (Riley et al. 2020). Consequently, much of the practical knowledge surrounding *Cannabis* cultivation and processing exists primarily in anecdotal sources such as online forums, blogs, and social media platforms. Despite their informal nature, these communities often comprise highly experienced cultivators whose insights offer valuable contributions to the emerging body of scientific knowledge. As global perspectives shift, primarily driven in part by growing evidence of *Cannabis*’s therapeutic potential (Helcman and Šmejkal 2022), there is increasing recognition of the need to formalize and validate these cultivation practices through empirical research. However, even in jurisdictions where medical cultivation is permitted, regulatory constraints continue to limit systematic investigation. One major consequence of this prolonged prohibition is the scarcity of peer-reviewed literature detailing standardized cultivation methodologies. Only recently has the scientific community begun to address this gap (Da Cunha Leme Filho et al. 2025; Krishnan and Yen 2025). This review contributes to that effort by examining the mainlining technique, a high-stress training method used to enhance bud yield through both biochemical and agronomic perspectives, and by evaluating its broader implications for *Cannabis* plant development and productivity.

### The molecular premise for mainlining

#### Apical dominance

The sessile nature of plants has promoted a rather advance ability to transform their branching architecture, above and below grounds as an evolutionary response that allows them to seek their essential requirements for survival including endogenous (phytohormones, ATP, genetic regulation, and mineral substrates such as nitrogen (N), potassium (K) and phosphorus (P) and exogenous (water, soil, CO_2_, light and space) needs. The branching process and its control are significant because they represent methods that allow plants to dominate aerial and subterranean regions. The genetic expression patterns of branching processes are driven by numerous variables, with minerals, water availability, light, and temperature being identified as the most influential (Li-Marchetti et al. 2015; de Jong et al. 2014; Pierik and Testerink 2014).

As a plant extends during growth, the apical meristems synthesize phytomers. These phytomers are a complex consisting of an internode, fixed to its axillary bud and one or more axillary buds. Triggering axillary buds can lead to two fates: sylleptic or proleptic where the former relates to the bud growing immediately after activation and the latter is a state of latency requiring external stimuli for bud outgrowth (Prats-Llinàs et al. 2019). Herbaceous annuals and woody dicotyledons are the most common examples of this latter two-stage development method, which has been linked to the developing apex's selective dominance over its axillary meristem. Apical dominance is now synonymous with this characteristic, which allows certain plants to develop in an avaricious manner while still having the ability to branch in response to environmental changes.

Apical dominance is upright growth localized in one main stem, which focuses on the development of the apical meristem rather than the auxiliary shoot meristems (Barbier et al. 2017; Kebrom 2017). *Cannabis* crops, like pines, sunflowers, and roses, are apically dominant plants (Wilson 2000). This dominance is closely linked to auxin and sugar production and consumption. Initially, it was considered that apical dominance was caused by the activity of Indole-3-acetic acid (auxins), a hormone generated in the shoot ends of the central axis and linked to plant height because it produces phototropic behaviour (Procko et al. 2016; de Wit et al. 2014). Auxin functions as a central signalling molecule in plant development, with its biosynthesis, transport, and perception extensively characterized in recent literature (Marzi et al. 2024; Brumos et al. 2018). It is predominantly synthesized in the shoot apical meristems (SAM) and immature leaves, where it contributes to organogenesis and meristem maintenance. Auxin transport occurs through a highly regulated, polar cell-to-cell mechanism, particularly along the stem vasculature. This directional movement is mediated by the asymmetric localization of PIN-FORMED (PIN) auxin efflux transporters, which facilitate basipetal flow. These are complemented by AUX1/LAX influx carriers, which enable auxin uptake into cells and help establish concentration gradients critical for developmental patterning (Vanneste et al. 2025; Ung et al. 2022; Teale et al. 2006).

Axillary buds remain quiescent due to the bidirectional transfer of auxins from tip to base. This assumption is supported by early investigations on auxin-induced stem elongation, which revealed a significant link between plant height and stem branching when an auxin-resistant 1 (axr1) variant of *Arabidopsis* was cultivated. Deng et al. (2012) discovered that inhibiting the expression of a tomato auxin signalling gene (S1IAA15) resulted in a significant reduction in plant height, as well as increased lateral side branching. Auxin is constitutively expressed in yucca plants, giving them an elongated phenotype. Auxins' delayed movement to the roots inhibits axillary bud development (Mason et al. 2014). This effect was demonstrated after auxins were applied to a decapitated stem, which prevented bud formation. However, applying auxins to axillary buds of decapitated plants did not result in bud inhibition, prompting doubts about the auxin-induced bud outgrowth inhibition concept. This inference was tested by radioactively tagging a tree stump with auxins and tracking its mobility after decapitation; interestingly, the auxins did not enter the axillary buds. Furthermore, once axillary buds were inhibited, auxin levels rose dramatically. Axillary buds are prospective plant development sites located at shoot nodes.

Decapitation, pruning, or horizontal animal grazing of primary SAM in the central axis of an apically dominant plant causes one or more axillary buds to emerge from quiescence, removing the correlative inhibitory effect and allowing plants to regenerate a new apex (Cao et al. 2023a, b). However, the auxin-induced apical dominance paradigm has been challenged. Apical dominance, however, has been linked to sugar availability rather than auxin supply, as previously supposed. Studies on the role of sugars in shoot branching in wheat, pea, sorghum, *Arabidopsis*, chrysanthemum, Rosa species, grapevine, and poplar have overturned our long-standing understanding of apical dominance and offers new avenues into the relationship between sugar and hormones and their effect on plant development (Gao et al. 2024; Beveridge et al. 2023; Schneider et al. 2019; Tarancon et al. 2017; Dierck et al. 2016; Mason et al. 2014; Van den Ende 2014; Kebrom et al. 2012).

Sugars are proposed as the limiting nutrient for apical development in contemporary versions of the classical apical dominance concept (Mason et al. 2014). Following decapitation, sugars travel long distances and accumulate in axillary buds during the bud release stage. The availability of sucrose has been shown to downregulate the expression of BRC1 (BRANCHED1), a key transcription factor that maintains bud dormancy. This downregulation facilitates bud activation and promotes outgrowth. In addition, sink organs in the central shoot, such as the stem and newly synthesized leaves, prefer sucrose to axillary buds. Axillary buds fail to initiate outgrowth when stem tissues act as dominant sink organs, effectively diverting assimilates and limiting sucrose availability. This physiological constraint reinforces the classical model of apical dominance, wherein auxin-mediated stem elongation indirectly suppresses bud activation by restricting the flow of carbohydrates essential for meristematic activity and growth (Coa et al. 2023; Beveridge et al. 2023; Bertheloot et al. 2020; Kebrom 2017; Mason 2013). Understanding apical dominance in *Cannabis* is critical for manipulating the entire plant architecture to maximize harvests.

**Fig. 1:**
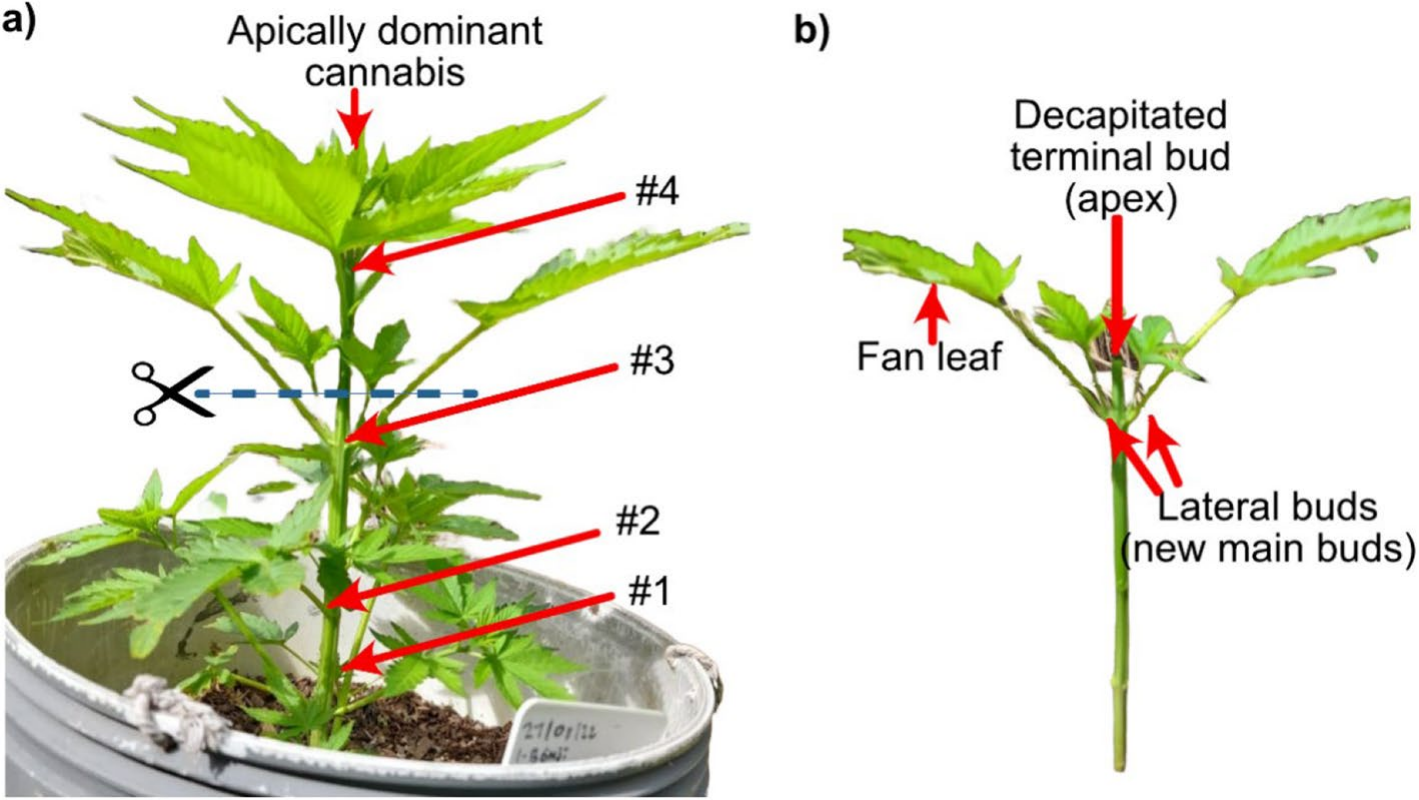
**a** Vegetating apically dominant *Cannabis* plant at week 4 of cultivation showing the development of four nodes; **b** Decapitation of apical meristem using scissors/knife/scalpel above #4 node

In Fig. 1, it is observed that energy appears focused on the top meristem. Here, the apically dominant *Cannabis* plant grows through phototropism, auxins allow the plant to extend upwards and favouring the main apical buds instead of the lateral buds. Allowing *Cannabis* plants to grow naturally can have a significant impact on yield, thus requiring more plants to reach the biomass weight target (Danziger and Bernstein 2021). Defoliating below the fourth node helps redirect energy and carbohydrate resources toward the upper canopy, particularly to support the development of axillary buds. This practice enhances light penetration and airflow. If the plant is not topped, it may grow too tall and develop a dominant central cola, which can be challenging for commercial cultivation due to uneven canopy structure and reduced lateral bud development (Caplan et al. 2018).

### Meristematic cell differentiation

Meristematic cell differentiation underpins the developmental plasticity of *Cannabis* and is central to its response to high-stress training. In plants, stem cells are maintained in specialized niches known as meristems, which include the shoot apical meristem (SAM), root apical meristem (RAM), and lateral meristems (LM). The SAM, in particular, is responsible for generating all aerial organs, including leaves, stems, and inflorescences throughout the plant’s life cycle (Hirano and Tanaka 2020; Aichinger et al. 2012).

**Fig. 2:**
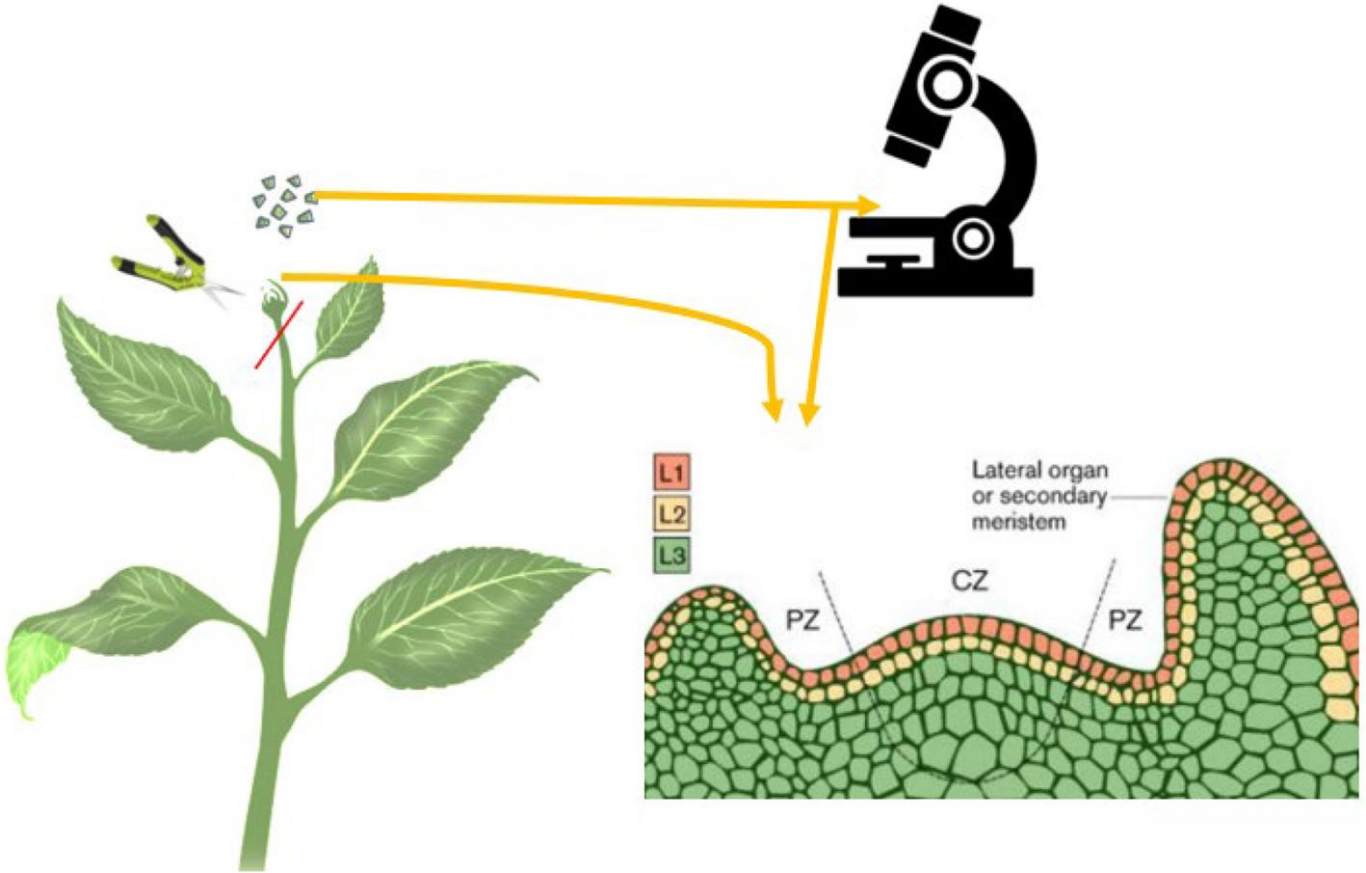
The apical meristem showing the outermost layer tunica, central zone (CZ), and peripheral zone (PZ)

The SAM is organized into distinct zones: the central zone (CZ), which houses slowly dividing stem cells; the peripheral zone (PZ), where cells differentiate into lateral organs; and the organizing center (OC), which maintains stem cell identity through regulatory feedback loops involving WUSCHEL (WUS) and CLAVATA3 (CLV3) (Yadav et al. 2013; Fletcher 2018). These zones are further stratified into three cell layers—L1, L2, and L3—each contributing to specific tissue types (Satina et al. 1940). An explanation and illustration are provided in Fig. 2.

Recent single-cell transcriptomic studies have confirmed that SAM stem cells exhibit low mitotic activity and maintain genome integrity while giving rise to differentiating progeny that form the plant’s architecture (Satterlee et al. 2020; Moreno et al. 2024). The dynamic balance between stem cell maintenance and organ initiation is regulated by transcription factors such as KNOX, WUS, and STM, as well as hormonal gradients involving auxins and cytokinins (Murray et al. 2012; Chang et al. 2020).

Understanding the regulatory mechanisms governing meristematic activity is critical for manipulating *Cannabis sativa* architecture through techniques such as mainlining, which rely on the precise modulation of SAM dynamics and the activation of axillary buds. The SAM maintains a population of pluripotent stem cells that give rise to lateral organs, while axillary meristems (AMs) form in the leaf axils and remain dormant or quiescent until triggered by developmental or environmental cues (Aichinger et al. 2012; Hirano and Tanaka 2020). Mainlining exploits this developmental plasticity by disrupting apical dominance—typically through decapitation—which reduces auxin flow from the apex and redistributes sugars and cytokinins to axillary sites, thereby promoting symmetrical bud outgrowth (Barbier et al. 2017; Beveridge et al. 2023).

The initiation and outgrowth of axillary buds are regulated by a complex network of hormonal signals, including auxins, cytokinins, and strigolactones, as well as transcription factors such as BRANCHED1 (BRC1), which integrates these signals to determine bud fate (Shimizu-Sato and Mori 2001; Wang et al. 2021). In *Cannabis*, this regulatory framework is particularly relevant, as short photoperiods and training interventions like mainlining induce architectural shifts that enhance inflorescence uniformity and yield (Spitzer-Rimon et al. 2019). By targeting the molecular and physiological levers of meristem function, mainlining enables growers to optimize canopy structure and maximize productive bud sites.

As illustrated in Fig. 2, SAM is typically organized into three distinct cell layers: L1 (epidermal), L2 (subepidermal), and L3 (internal tissue layer contributing to vascular and stem tissues), although this stratification can vary across plant species (Satina et al. 1940). These layers are part of the tunica-corpus organization, where the tunica (L1 and L2) undergoes predominantly anticlinal cell divisions to maintain surface integrity, while the corpus (L3) divides in multiple planes to contribute to internal tissue development.

Within the deeper L2/L3 layers lies the organizing center (OC), a critical domain that maintains stem cell identity by regulating the activity of the overlying central zone (CZ). The OC achieves this through the expression of key transcription factors such as WUSCHEL (WUS), which moves from the OC into the CZ to promote stem cell fate and inhibit differentiation (Lindsay et al. 2024). This signalling is tightly controlled by feedback loops involving CLAVATA (CLV) genes, which restrict WUS expression to prevent over proliferation of stem cells, thereby maintaining a balance between stem cell maintenance and organ initiation.

### Strengthening the vascular bundles

#### Spatial organization of vascular bundles

Plants belonging to the *Cannabis* genus are herbaceous dicotyledonous plants that differ from monocotyledonous plants in that they possess vascular tissue. The vascularization of plants is an evolutionary advantage that facilitated the widespread colonization of plants on land (Carteni et al. 2014). The response to mainlining is a broad canopy. It has been observed that repeated apical decapitation may lead to increased stem thickening, likely as a result of the plant reinforcing structural integrity in response to stress training. However, the relationship between stem diameter and the number of decapitations is not yet well-characterized, requiring further empirical investigation.

Upon the removal of an apical bud, the plant interprets this event as a mechanical stress signal, triggering the activation of adaptive developmental pathways. In response to this injury-induced cue, a cascade of biochemical signals, including phytohormones and reactive oxygen species, is initiated. These signals promote the synthesis and translocation of regulatory compounds that activate stem cells within the meristematic zones, facilitating vascular tissue regeneration and compensatory growth. (Zhang et al. 2023; Lee 2018). The plant's vascular system, which extends throughout the whole plant body, has conventionally been recognized for its dual functions: a) facilitating the movement of water, nutrients, hormones, and signalling agents along the apical or basal axis, and b) offering mechanical support and strength (Ruonala et al. 2017; Lucas et al. 2013). The vascular system develops as a result of the combined action of hormones, axillary regulatory substances, their corresponding receptors and transporters, and the numerous temporo-spatial regulators of gene expression, indicating a tightly coordinated regulatory process.

The separation of a *Cannabis* plant axil reveals two distinct levels of spatial structure within the vascular system. *Arabidopsis* models gave deeper insights into plant vasculature, revealing the presence of five to eight vascular bundles. Nonetheless, the initial formation of vascular bundles suggests that they form a modular chain oriented radially (Smet and De Rybel 2016). Radial patterns are facilitated by transcription factors (TFs) such as KANADI (KAN) and class III HD-ZIP (PHABULOSA (PHB), PHAVOLUTA (PHV), REVOLUTA (REV), and CORONA (CNA), which operate as the main regulators of radial patterning. The antagonistic participation of HD-ZIP III and KANADI in radial patterning has been demonstrated, whereby they contribute to the delineation of differentiation at the abaxial and adaxial domains necessary for the appropriate development of vascular tissue (Satterlee and Scanlon 2019; Ilegms et al. 2010).

According to Ruonala et al., (2017), Smet and De Rybel (2016), Yoshida et al. (2014), and Lehesranta et al., (2010), the composition of vascular systems consists of highly organised transporting tissue types, known as xylem (wood) and phloem (bark), together with less-known intervening procambial (cambial) cells of pluripotent status that are fated to differentiate into various xylem and phloem structures. The determination of cell identity in these tissues is specific to the requirements of the plant. The process of primary vascular organogenesis involves the division of vascular cells, followed by the formation of vascular patterns, identification of vascular cell identities, and ultimately the differentiation of vascular cells (Ohashi-Ito and Fukuda 2017). Inducing embryogenesis in vascular meristems, stress triggers mitosis of stem cells, leading to an augmented stem cell population that can either remain undetermined or differentiate into the absent xylem (center) and phloem (periphery) tissue centers. These tissue centers collaborate to facilitate the healing and restoration of pruned sites (Jouannet et al. 2015; Miyashima et al. 2013).

Plant vascular structures, xylem and phloem, originate from vascular meristems that become active after primary organ differentiation. These meristems form a larger group of lateral meristems, together with cork cambium, which contribute to the formation of gymnosperm bark (Miyashima et al. 2013). The intricate nature of the differentiation process of cambial cells into xylem and phloem has resulted in a scarcity of studies elucidating the molecular regulatory pathways in diverse plants throughout the kingdom. Stem cell differentiation tracking in diverse contexts is a continuous and urgent pursuit, facilitated by modern technology that enables the modification of multiple biomolecular pathways involved in plant survival and productivity (Chang et al. 2022; Randall et al. 2015). Contemporary genetic and molecular approaches have enhanced our comprehension of the regulatory mechanisms for vascular development, which was previously based on *Populus* and *Zinnia* models.

### Stress, injury, response, and restoration

How a *Cannabis* plant responds to injury caused by high-stress training and the subsequent coordinated strengthening of its stem is determined by the precise spatial and temporal arrangement of key stem cells that provide cues for renewal of the vascular threads required for plant support (Lehesranta et al. 2010). Despite their evident relevance, vascular systems are a relatively underutilized plant system. Interestingly, the focus is on the hormonal (Vanstraelen and Benkova 2012) and genetic components (Caño-Delgado et al. 2010) that participate in organ developmental regulation. Additionally, the critical role of auxin movement towards developing organ arrangement is more investigated, with the environmental stimuli implicated in this process often ignored. Overall, research efforts have been hampered by the difficulty of tracking vascular systems due to their embedded nature in plant structure (Kondo et al. 2016; Peng et al. 2013), which limits access and prospection of these regions.

When a *Cannabis* plant experiences apical cleavage, xylem and phloem tissues differentiate into various cell types, including xylem strands, xylem parenchyma cells, and tracheary elements (TEs) that make up the vessels and tracheids that will eventually constitute the secondary cell wall (SCW) (Ohashi-Ito and Fukuda 2014). These elements are regulated in the stem cell niche by apoptotic processes (Rajagopalan et al. 2024). The combined action of the xylary strands and TEs results in a thicker and more robust SCW, which is the principal component of the cellulosic biomass (fibre) that is unique in its application for biofuel generation (Carpita 2012; Demura and Ye 2010). SCW is the region in plants where synthesized lignin polymers are deposited as a protective layer to prevent harmful substances from entering the cells (Kumar et al. 2016). Lignified cells are repeated xylem subunits that are stiff and resistant to water pressure and mechanical stress (Oda and Fukuda 2012). These cells allowed for the establishment of larger plant designs while maintaining stability (Lucas et al. 2013), and they are recruited in the case of stress or injury to restore plant development. Earlier research into genetic manipulation of SCW production and vascular differentiation indicated that they are mediated by the same signaling network (Turner et al. 2007).

When plants are topped and their biomass doubles, yield improvements may be due to the effect that vascular differentiation has on SCW synthesis. Phloem is formed through the coordination of sieve tube elements, companion cells, phloem strands, and phloem parenchyma cell types. Phloem synthesis is facilitated by genetic markers such as altered phloem development (APL), SUPPRESSOR OF MAX2 1-LIKE3 (SMXL3), SMXL4, and SMXL5 (Agusti and Greb 2013; Roszak et al. 2021). The transcription factor SMXL5 is involved in phloem and distal cambium cell differentiation, which promotes secondary phloem growth in the radial stem. The TF, SMXL5 is implicated in phloem and distal cambium cells differentiation to enhance secondary phloem growth in the radial stem (Wallner et al. 2017, 2020; Shi et al. 2019). All these cell types, both xylem and phloem are derived early during embryogenesis from a select few procambium progenitor cells (Smet and De Rybel 2016).

In vascular plants, procambial cells divide into two types that influence the expression of all major plant tissue types: anticlinal and periclinal. An anticlinal or perpendicular cell division (AD/PD) is the process of adding a new cell to an existing cell file, which drives longitudinal development along the plant's central axis. Periclinal or parallel cell division (PD) increases the number of cell files and contributes to radial development (Smet and De Rybel 2016). These divisions produce offspring cells of varying functions and sizes (De Smut and Beeckman 2011). The spatial arrangement and relative frequency of procambium, phloem, and xylem suggest continuous differentiation that varies greatly and is unique to each species and organ within a population (Agusti and Blazquez 2020; Carteni et al. 2014). This impact can also be observed in plant tissues, where the organization and relative frequency of all the many cellular structures that make up the xylem and phloem are distinctive by species and organ within a species. It is for this reason that ecotypes (subspecies adapted to a certain set of environmental stimuli) exhibit some variance in such traits (Adams et al. 2023).

Procambial differentiation into vascular structures is thought to be a hormone accumulation event triggered by auxins acting on polar-transport pathways. Experiments demonstrated that exposure to the auxin reporter DR5 as well as the AtHB8- a pre-procambial marker generated by auxin, resulted in the differentiation into vascular strands in the leaves (Biedroń and Banasiak 2018). PIN1, an auxin channelling agent, transports auxins to the plant's provascular zones to assist in vascular synthesis (Ung et al. 2022; Zhou and Luo 2018; Lehesranta et al. 2010).

*Cannabis* plants exhibit remarkable developmental plasticity, enabling dynamic reconfiguration of their vascular systems in response to high-stress training (HST) techniques such as decapitation, super cropping, and mainlining (Mazur and Friml 2017; Agustí and Greb 2013). These stress-induced modifications activate vascular stem cells and initiate compensatory differentiation processes that support structural reinforcement and resource redistribution. However, the molecular and biochemical mechanisms underlying these vascular responses remain insufficiently characterized. A key area of inquiry involves whether real-time tracking of vascular bundle differentiation during training could inform the development of novel cultivation strategies aimed at producing phenotypes with enhanced productivity and resilience.

Notably, the response to HST may vary across *Cannabis* subspecies. For instance, *C. sativa* var. *sativa* and *C. indica* exhibit distinct architectural and physiological traits, suggesting species-specific vascular adaptations to training. Comparative studies on vascular differentiation across these taxa could yield insights into genotype-specific optimization of training protocols. Previous research has primarily focused on signal transduction and intercellular communication within vascular tissues, emphasizing the role of hormonal gradients, transcriptional networks, and tissue-specific crosstalk in regulating vascular development (Hunziker and Greb 2024; Ma et al. 2023; Agustí et al. 2020; Notaguchi and Okamoto 2015; Lehesranta et al. 2010). Expanding this knowledge to include *Cannabis*-specific models will be essential for translating physiological responses into targeted agronomic interventions.

### Canopy management and hyperproduction of colas

*Cannabis* plants will flower naturally in the absence of human influence. The link between vegetative growth (biomass production) and cola/trichome production (THC/CBD) has long been recognized. Traditionally, abundant yields and plant vigour were achieved by manipulating many agro-edaphic elements such as light, humidity, and temperature to trigger changes in biomass accumulation in order to produce high-quality products (Ačko et al. 2019). Recently, training approaches, particularly pruning and defoliation techniques, gained prominence as strategies for manipulating *Cannabis* tree canopies (Zhang et al. 2021). Mainlining, as a technique, can be considered a type of canopy management. Mainlining is also a method for controlling the amount of incoming radiation and the percentage of radiation intercepted by the plant's canopy. One of the fundamental benefits of mainlining is that *Cannabis* crops are trained to build an even canopy, with the understanding that the management of the architecture of the canopy formed is critical to higher bud output (Murchi and Burgess 2022; Anthony and Minas 2021). Therefore, canopy management refers to the manipulation of *Cannabis* plants to maximize the production of high-quality buds and seeds (Adiga et al. 2020). The amount of light that enters the canopy determines the morphology of the *Cannabis* tree (Moher et al. 2022; Magagnini et al. 2018). The goal of canopy management is to efficiently re-route carbon delivery between these developmental organs while minimizing interference with development and growth. In *Cannabis* production, the volume, size, and orientation of the stem and branches (bending) determine the canopy structure (Pandey, 2021; Tang et al., 2019).

A canopy's density (compaction) is mostly determined by the size and number of leaves (Tang et al., 2019). The training and pruning process determines the density, and this is impacted by the ratio of incoming radiation to intercepted radiation (Vishal et al. 2020). *C. sativa* var*. sativa* spp. plants have a spread and intermittent canopy with thinner leaves and stems. *C. sativa* var. *indica* spp. on the other hand, has a flatter and level canopy due to its compact leaf arrangement and sturdy appearance. Despite these structural differences, both subspecies tend to grow rapidly when left uncontrolled. An unmanaged tree canopy has a significant impact on *Cannabis* bud mass and organoleptic characteristics (Tang et al., 2019). Where mainlining excels is in its ability to significantly reduce the size of canopy shading by adjusting *Cannabis* plant height for improved light interception and penetration (Adiga et al. 2020), effectively diverting carbon resources to lateral buds and morphing the plant's phenotype from a slender, straight plant to a bushy, more uncontrolled development of shoots and stems (Tang et al., 2019; Zhang et al., 2021). In this case, mainlining represents an innovative method to tame the uncontrolled state of *Cannabis* plants to a controlled and directed level of nutrient allocation, resulting in an even canopy that allows for maximum land productivity and climatic conditions that accommodate optimal outputs in terms of yields and quality in a three-dimensional setting (Rodge et al. 2023; Adiga et al. 2020). Other plant parts can be used to maintain the canopy, but controlling blooming onset and bud expansion is less prevalent (Wani et al. 2021).

Implementing effective management of *Cannabis* canopy structure throughout the vegetative and blooming phases of the life cycle offers several benefits. The technique guarantees optimal light use by ensuring that buds have the same photoperiod, in contrast to uneven tops that compete for sunlight (Kumar and Sharma 2020). These phenomena can be attributed to the remarkable absorption and light dispersion mechanism of the canopies. Increased numbers of topping or pruning can result in higher biomass output and are the main advantage of canopy management, as they facilitate the capturing and conversion of light by smaller trees compared to larger ones, thereby improving production efficiency due to photosynthetic conversions (Ahsan et al., 2024; Chen et al., 2024; Rodge et al., 2023; Uselis et al., 2022; Lanoue et al., 2022; Tang et al., 2019). The achievement of a uniform canopy facilitates the provision of structural support for larger *Cannabis* plants. Notably, canopy preservation enables the proper development of secondary and tertiary branches. The phenomenon of shade becomes significant in instances of sunburn and is a prominent characteristic in the management of canopy. Management of inter-cultural activities pertaining to pests and diseases is facilitated by maintaining colas at an equal distance from the main crown, so preventing foliage overlap (Fuentealba-Sandoval et al. 2021). This arrangement enables enhanced air ventilation around the plant, hence enhancing the efficiency of fertilizer spraying. Additionally, the lateral buds provide a larger surface area for absorption of light radiation (Matzneller et al. 2022; Peavy et al. 2020).

Colas are the name for Cannabis inflorescence. In certain instances, it is referred to as the terminal bud at the plant’s apex. In untrained, healthy feminized plants, a single dominant apical cola typically develops at the terminal bud, while smaller axillary colas emerge along lateral branches (Spitzer-Rimon et al. 2019). The process of trimming, topping, and structured training growers can manipulate plant architecture to promote multiple primary bud sites. These formerly secondary sites are redirected to receive equal hormonal and nutrient support, resulting in symmetrical and evenly developed colas (Spitzer-Rimon et al. 2019). When the apical meristem is removed via topping, the plant undergoes a physiological response known as ‘apical dominance disruption’. This process involves the redistribution of auxins, cytokinins, and sugars, which activates lateral meristems and induces bifurcation at the node below the cut site. The result is the emergence of two new primary shoots, each capable of developing into dominant colas with comparable morphology, bud density, and terpene profiles (Beveridge et al. 2023; Gaudreau et al. 2020). This regenerative mechanism reflects a well-documented botanical strategy for maximizing reproductive potential and canopy exposure following mechanical stress. When executed correctly, this approach enhances uniformity across flowering sites, streamlining harvest operations and reducing the prevalence of underdeveloped “popcorn buds,” which often result from poor light penetration and intra-canopy competition (Spitzer-Rimon et al. 2019). Mainlining further synchronizes ripening by optimizing light distribution and photosynthetic efficiency across the canopy. This technique improves resource allocation, leading to enhanced bud development, uniform maturation, and increased yield quality.

Canopy management practices, such as selective pruning (dormant, summer, and root pruning), training, scoring, spraying, girdling, rootstock selection, and raceme harvesting, are integral to maintaining vegetative balance and maximizing reproductive output, particularly in cultivars with extended growth cycles. These methods also offer a cost-effective strategy for managing plant vigor and ensuring synchronized ripening of inflorescences due to enhanced photosynthetic rates (Rodge et al. 2023; Wani et al. 2021; Mitra et al. 2017). This characteristic feature of mainlining of doubling following each decapitation event is attractive to cultivators. The quantity of toppings on *Cannabis* plants is dictated by the genetic composition and overall strength of the strain. To achieve optimal results, it is typical to execute 3 to 4 topping exercises during the vegetative phase. It is also possible to attain 5 toppings, where 32 colas develop, which is regarded as the threshold for achieving the highest yield that does not compromise plant containment capacity. Once the number of tops exceeds 6, the plant may exhibit symptoms of stress that may be difficult to resolve. To be able to achieve exponential growth in production which is the primary objective of canopy management and to ensure equal amounts of sunlight reach these colas; the colas are arranged in a manifold configuration, with a plant ring utilized to divide the main stem into two identical colas, allowing the plant to be placed evenly to maximize capture of sunlight (Haze 2024). This manifold facilitates a uniform dispersion of nutrients and enhances the formation of cola along the manifold. This manifold can be conceptualized as a branched vascular conduit system that facilitates efficient nutrient distribution throughout the plant. Each bifurcation within this system enhances the spatial arrangement of stems and leaves, improving light penetration and photosynthetic exposure. This architectural optimization contributes to the development of higher-quality fruits by ensuring uniform resource allocation and canopy openness (Caplan et al. 2018; Trancoso et al. 2022).

### Eliminating unnecessary growth and enhancing active bud sites

*Cannabis* plants in the wild that are untrained assume a bushy and branched out appearance, running the full length of the stem. These stems and branches naturally develop flowers and leaves that are stacked where they start at the bottom and bulge at the top, similar to the structure of firs (Christmas trees). All sections of the *Cannabis* plant can produce buds and resin; however, the position at which these bud sites are stationed along this fir-structure becomes cogent in terms of bud yield and quality. This stacked arrangement is not ideal for the lower branches of the plant (Lentz et al. 2023).

In controlled indoor cultivation of *Cannabis*, the distribution of photosynthetically active radiation (PAR) is often limited. Light is typically concentrated in a fixed position, resulting in the upper inflorescences receiving most of the light and energy required for development into large, dense floral structures (Rodriguez-Morrison et al. 2021). In the lower bud sections of the plant, less PAR is received or is simply out of the range because of increased shading, leading to suboptimal development of this region (One 2023). In this region, plant leaves convert chlorophyll into carotenoids, leading to the senescence of some leaves (Wang et al. 2020). If the lower section remains untouched, these lower branches and nodes sap the plant’s resources and energy, causing the resultant buds to be devoid of aroma, flavour, and potency. The outcomes are observed to be an inferior size, density, flavour, and in general, an unappealing appearance compared to the buds with better light exposure on the upper canopies (Tarafder et al. 2023).

*Cannabis* growers over the years, understood that eliminating this unnecessary growth below the main (active) bud sites can encourage a transfer and concentration of the plant’s energy (resources) from the lower branches to the upper active bud site canopies that are more receptive to light. For this reason, the training technique of “lollipopping” gained prominence in the *Cannabis* cultivating communities as a canopy management strategy towards addressing the challenge of suboptimal bud development (Danziger and Bernstein 2021; Voser 2020).

Lollipopping is an aggressive form of plant high-stress training that aims to refocus the energy resident in *Cannabis* plants by removing all lateral branches, nodes, and fan leaves from the bottom third of the plant that do not contribute to *Cannabis* bud development and redirecting it to the areas of the plant that receive the most sunlight, the upper canopy (Trancoso et al. 2022; Danziger and Bernstein 2021). The goal of this technique is to thin the plant's basal portion, with branches completely naked below and growth beginning only at the primary bud sites (One 2023). As the plant grows, a large top-heavy canopy forms above a bare stem with the morphology of a lollipop, hence its unusual name (One 2023; Wei et al. 2022). Despite its contemporary classification, lollipopping is a variation of lower pruning.

The approach is suitable for both photoperiod and autoflowering strains. Eliminating unnecessary plant development in outdoor cultivation does not result in a positive net-outcome because this type of cultivation is naturally dependent on the movement of the sun and the preeminent penetrating power of its rays (Wei et al. 2022). However, lower buds that are known to be covered with detritus should be removed. This approach should be used in conjunction with pruning; while they are the same thing, pruning is intended to remove long shaded branches that have grown excessively thick (Danziger and Bernstein 2021). *Cannabis* subspecies can react differently to lollipopping, for example, since *C. indica* var. indica spp. and *C. sativa* var. *sativa* spp. types have various development patterns and life cycles; it would imply that a bespoke lollipopping regime that addresses these differences should be developed for each. Furthermore, like with most high-stress training methods, this method can temporarily inhibit plant development during the energy redirection phase (Wei et al. 2022). To mitigate this effect, lollipopping should be done near the end of its vegetative cycle, preferably a week before flowering begins (Trancoso et al. 2022).

Pruning the lower part of *Cannabis* plants redirects nutrients to the top sections of the canopy, which has numerous benefits (Ashraf and Ashraf 2014). Lower branch trimming and defoliation offer adequate light coverage to active buds at the top of the canopy, resulting in higher overall growth and resin (trichome) production (Kumar and Sharma 2020; Voser 2020). As the plant's energy and resources accumulate in the upper canopy, it exhibits increased vertical growth while also increasing bud size, density, and flavor. The yield is determined by the amount of light entering, the air velocity around the buds, and the distribution of nutrients around the plants (Thakur et al. 2018; Verma and Shukla 2015) and it is a significant advantage for indoor production to generate consistent buds that are larger, heavier, and more potent than untrained plants (Voser 2020; Mitra et al. 2017). Lollipop plants have a lower susceptibility to pests and diseases because of enhanced airflow within the canopy (Danziger and Bernstein 2021). Ventilation reduces the likelihood of mold, mildew, and other diseases clinging to important plant organs that would otherwise have to be destroyed, increasing crop loss (Akpenpuun et al. 2023). Furthermore, laborious cultivation duties such as watering, pruning, and pest management become easier with this HST method due to a lack of branches and foliage interference, allowing producers to divert time and care to other critical components of the growth process (Haze 2024; Sholl 2020).

Other canopy management strategies that have been described in the *Cannabis* community are defoliation and “schwazzing”. A “Schwazze” is an extreme defoliation technique, where the entire fan leaves are removed during the flowering stage. The removal of these leaves refocuses the plant energy towards flowering (Bergman 2023). If the plant is left on its own devices, it’s a slow process where the fan leaves, which contributed biomass and accumulated during vegetation, then lose their colour from green to yellow once the leaves has transferred into energy into the bud sites, eventually causing a shrivelled appearance (Dias 2024). Therefore, their active removal efficiently facilitates the transfer of energy and enhances flower production. This technique relies on the grower’s experience (Gorden and Owens 2024).

## Mainlining of Cannabis plants

### Methods and techniques

Mainlining is a series of techniques that exploit the understanding of apical dominance in plants. Mainlining combines low-stress training (stem bending or tie-down and Screen of Green (SCROG) and high-stress training methods (apical fimming and topping, lollipopping, basal pruning, and supercropping) to split the main colas of *Cannabis* plant for enhanced yields (Danziger and Bernstein 2021; Trancoso et al. 2022). In mainlining, a manifold by decapitation of the main central axis is created early in the growing season to break the apical dominance (see Fig. 1a and b). Here, the Y-shaped manifold distributes hormones, nutrients and water to each branch equally, leading to a system of uniform cola-bearing inflorescence with equal height, girth, and distance (canopy). As a consequence, inflorescence yields are drastically improved with less space and is a method that confers control to the grower as it mainly relies on sensible pruning instead of sophisticated farming technology (Haze 2024; Spitzer-Rimon et al. 2019).

The removal of the primary stem allows lateral buds to grow new branches due to increased exposure to sunlight (Trancoso et al. 2022). The plant is manipulated to distribute auxins, cytokinins, strigolactones, energy, and nutrients to all its parts, rather than concentrating these resources in a single central cola, so promoting the growth of larger and more abundant final yields (Lentz et al. 2023; Ačko et al. 2019). Mainlining ensures uniform solar exposure across all plant levels by expanding the canopy, thereby facilitating the growth of smaller, lighter flowers at the plant's base (Dias 2024). Symmetry is a crucial aspect of mainlining (Sholl 2020). Plants uniformly distribute auxins as metabolic pathways often exhibit a linear trend (Ung 2022). Failure to adhere to fundamental principles of symmetry can lead to unbalanced plant growth, resulting in an uneven canopy that receives inconsistent light intensities, which impacts overall production (Sholl 2020; Hillock and Schnelle 2017).

Clonally propagated industrial hemp is unsuitable for mainlining due to its thinner root structure, which renders it susceptible to edaphic and anthropogenic stress associated with the training procedure (Dreger et al. 2023; Martinkova et al. 2020). Additional caution is necessary while topping nodes. Moreover, clones have a lack of apical dominance and may frequently present asymmetry (Campbell 2019). Seeds can yield a plant exhibiting single cola (inflorescent) dominance; however, clones may display a divergent structure, leading to an uneven distribution of seeds and flowers (Asghar et al., 2023; Zarei et al., 2022). The advantages of mainlining are more evident when seeds serve as the propagation medium. This is why the benefits of mainlining are more pronounced when seeds are used as propagating material. This is because, a) seedlings have higher chance of survival during training as they naturally form a decent root size and allows for errors especially to tyros and b) Seedlings are more likely to be more vigorous and symmetrical (Asghar et al., 2023; Grossnickle and Ivetić, 2022) Table 2 summarizes the advantages and disadvantages of mainlining as a technique.

**Table 2:** Advantages and disadvantages of mainlining technique (Trancoso et al. 2023; Dreger et al. 2023; Sholl 2020; Ačko et al. 2019)

Advantages	Disadvantages
Maximises space	Experience and expertise required
Increases cola count and quality manifolds	Not suitable to some strains of *C. sativa*. var sativa and especially autoflowering strains
Improve plant vegetative vigour	Involves intensive labour for its initiation
Easy maintenance and harvesting (less defoliation)	Permanent plant damage and reduction of yield due to use of wrong technique
Alteration of tall, stretchy strains to stout, bushy structures	Risk of infection at wound site
Reduction of pathogens (Mildew and Mold)	If done late, plant may not have enough resources for recovery and regeneration
Obtain an even canopy = higher and even light absorbance and air circulation throughout	Increases the vegetative state of the plant resulting in delayed flowering (~ 2 weeks)
Applicable to indoor and outdoor cultivations	

In plants, apical development occurs as a consequence of the plant pursuing the path of least resistance in its growth. Apical development alone does not inherently result in improved yield; rather, it is the modification of plant structure that facilitates enhanced yields (Trancoso et al. 2022). By inducing the central apex of a plant to grow horizontally, parallel to the ground, the plant perceives that its principal cola (main auxin site) has been severed, prompting it to enter a survival mode and seek alternative pathways for further development (Ačko et al. 2019). As the primary stem develops, the effect of apical dominance diminishes. The influence of auxins is more significant at the root tips; yet lateral buds and branches develop basally without inhibition (Cao et al. 2023a, b; Schneider et al. 2019). In this instance, apical dominance is diminished, facilitating the elongation and growth of lateral buds (Kebrom 2017).

**Fig. 3:**
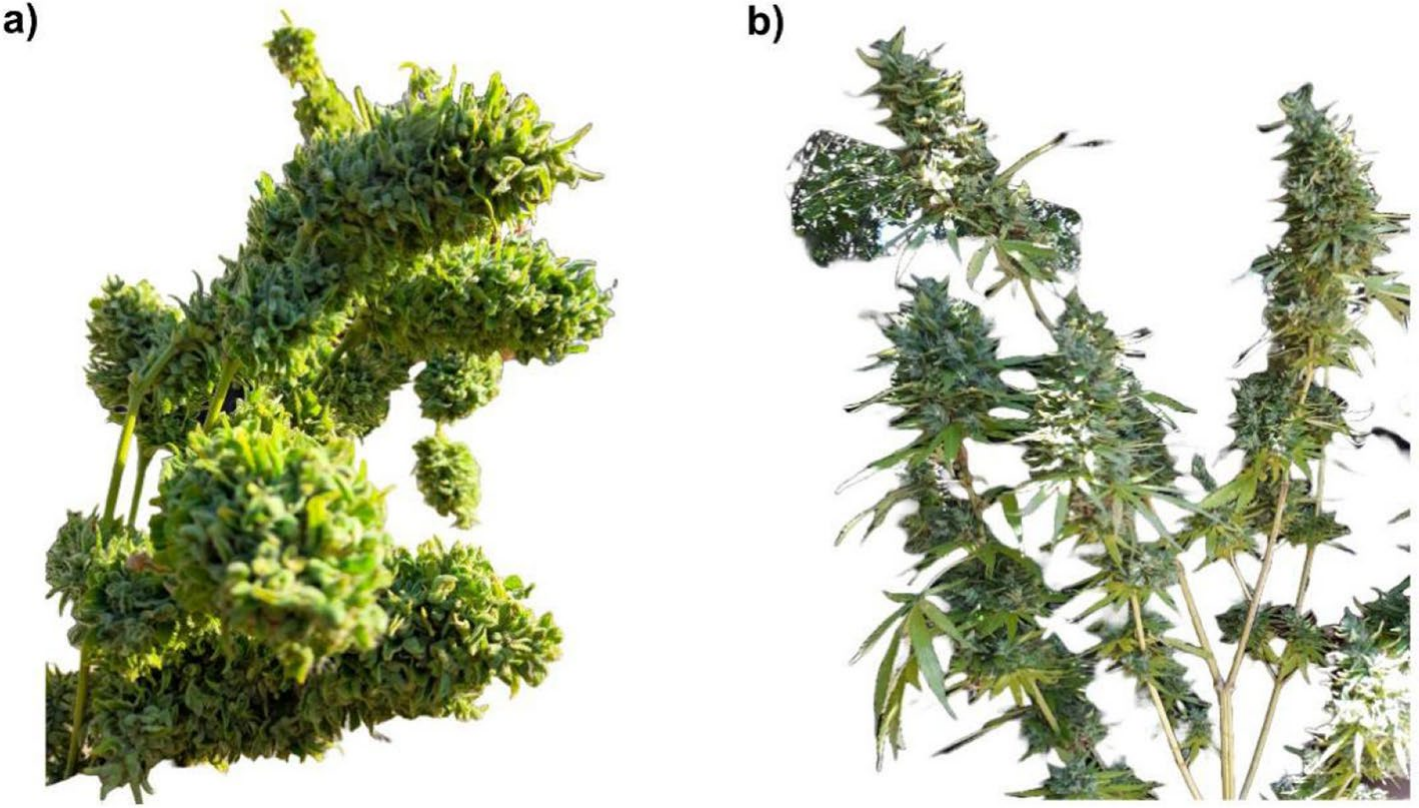
**a** *Cannabis* plant that experienced mainlining, resulting in increased cola count and overall yield, with enhanced biomass. **b** *Cannabis* plants without mainlining, inflorescences (buds) are smaller and less compact

In mainlining, the quantity of apical pruning dictates the number of colas, and the correlation between topping and the number of colas is linear (Lentz et al. 2023; Mazzoni et al. 2022) The repetition of the split, along with supplementary and consecutive topping procedures, enhances the manifolds and doubles the quantity of inflorescences (Lentz et al. 2023). One top produces two colas; two tops yield four colas; three tops generate eight colas, and so on. Topping is typically performed to achieve 8 (× 3) or 16 (× 4) colas, contingent upon the pruning series employed (Haze 2024). Figure 3 is a feminized photo-period *Cannabis* plant which started with a singular primary cola; however, mainlining enables the production of 2, 4, 8, 16, 32, and/or 64 robust inflorescences (THC/CBD) or seed-laden colas per plant. The primary feature of mainlining is its use of spatial limitations to support *Cannabis* plants.

It is important to note for Fig. 3a that even though the bud density may appear improved due to uniform canopy exposure, the primary benefit of mainlining lies in yield optimization rather than direct enhancement of individual bud density. For Fig. 3b, *Cannabis* plants without mainlining tend to produce fewer colas, resulting in overall smaller yields. Although both plants were topped, the plant buds without mainlining appear less uniform due to uneven light exposure.

**Fig. 4:**
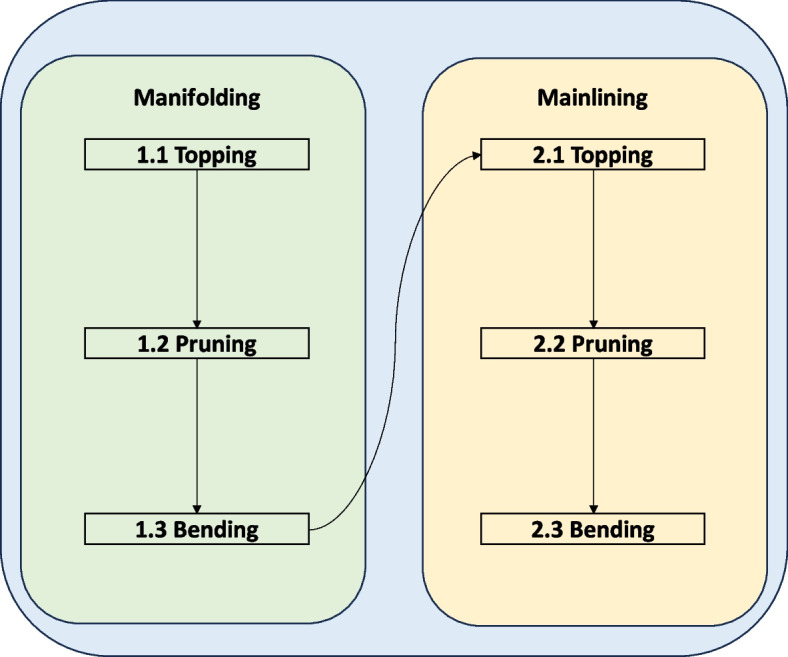
Process flowchart of mainlining technique

Research on plant responses to apical bud removal has documented this occurrence across various plant species, particularly angiosperms such as roses and *C. sativa* (Beveridge et al. 2023). Certain plants, including ornamental shrubs, grapevines, and field crops such as tomatoes, cucumbers, pumpkins, melons, paprika, and aubergines, are frequently pruned to enhance output (Filipov et al. 2021; Marini et al. 2020; Zrar et al. 2019; Liashenko et al. 2018; Mourão et al. 2017). The most suitable cannabis cultivars for mainlining are those with strong apical dominance, symmetrical growth, and medium to tall stature with minimal lateral branching. These traits support uniform canopy development and efficient light distribution. Indica-dominant hybrids and select phenotypes of *Cannabis sativa* with stable internodal spacing are often preferred due to their structural predictability. In contrast, pure *C. sativa* varieties tend to exhibit excessive lateral branching and longer internodes, making them less ideal. Autoflowering strains are generally unsuitable because of their short vegetative phase and limited responsiveness to high-stress training techniques. However, the phenotypic plasticity of *Cannabis sativa* allows for some adaptability across genotypes. With careful timing and environmental control, even less conventional strains can be moderately trained using mainlining methods (Campbell et al. 2019; Bully Seeds 2023; Horn Creek Hemp 2024) (Fig 4).

### Considerations in performing mainlining

Although yields are enhanced with mainlining, the resulting changes in the metabolic pathways involved remain obscure (Trancoso et al. 2022). Moreover, other challenges linked to the absence of standardized techniques make it difficult to determine the overall efficiency and safety of this technique, as it amalgamates previous LST and HST methods (Marcu and Schechter 2025). Furthermore, there is a lack of consensus on its application, and it has not been comprehensively detailed in academic literature. This gap has been noted by Caplan et al. (2018) and remains sparsely investigated in subsequent peer-reviewed journals, underscoring a critical gap between cultivation practices and scientific research within the context of mainlining. Much information regarding mainlining is derived from blogs and personal cultivation experiences, primarily focusing on feminized or autoflowering strains. Research in the future may also need to re-imagine using mainlining for the hemp variety, considering the yield output benefits.

**Fig. 5:**
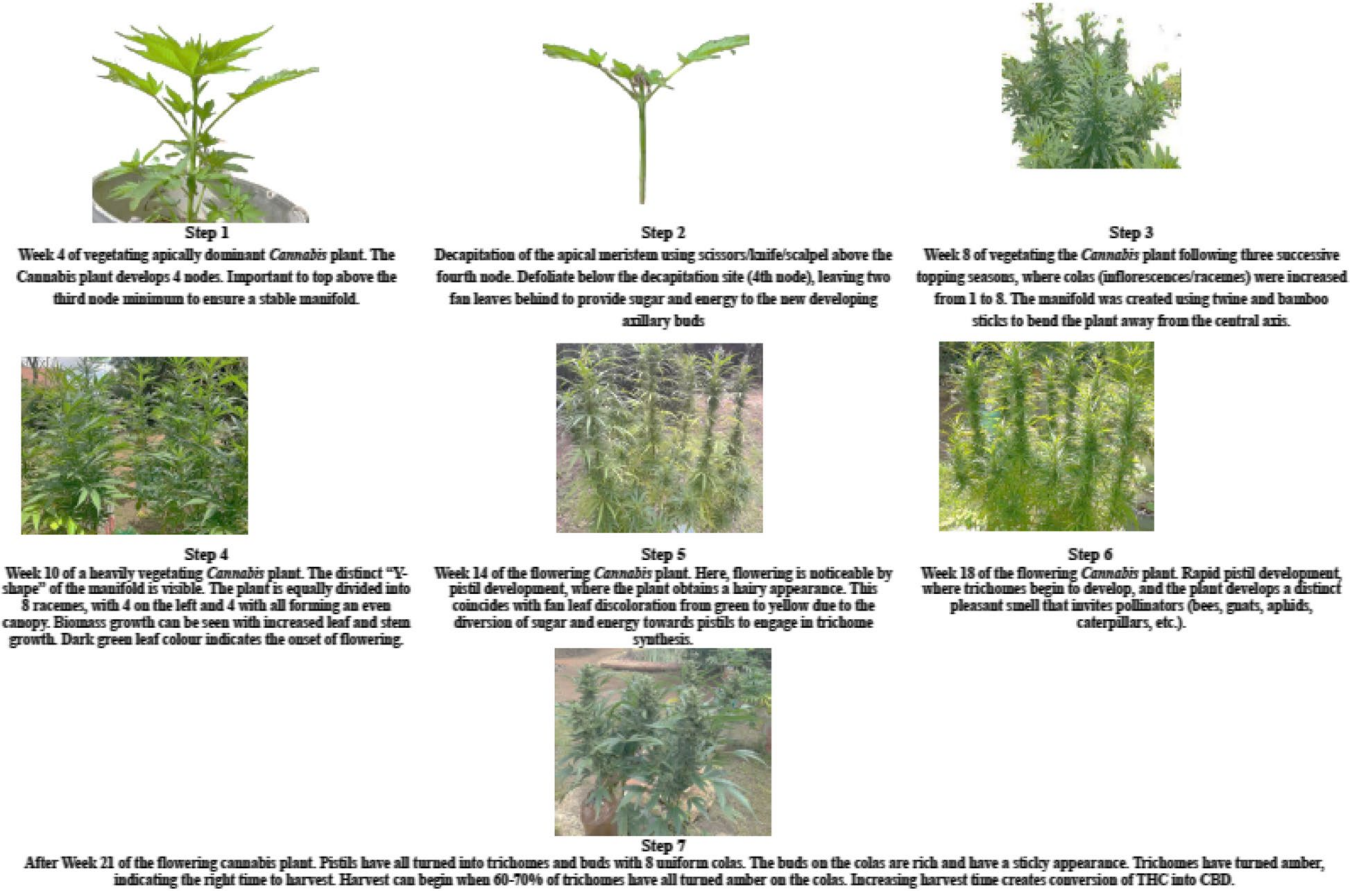
Picture representation of the series of steps in the mainlining technique

A summary of the sequence of mainlining techniques as adapted from NetraManjunath (2025); Haze (2024), Khan (2024), One (2023), Danziger and Bernstein (2021), Voser (2020), Spitzer-Rimon et al., (2019) is as follows:

1. Mainlining poses a risk of disease and infection to plants due to the recurrent trimming required in the technique. Compliance with aseptic methods is essential in mainlining. Hands must be cleansed with soap and alcohol to ensure sterility. Tools utilized for defoliation and topping (such as pruning shears, razor blades, scalpels, pocketknives, etc.) must be sterilized with alcohol and direct flame to minimize the risk of the transfer of infection.
2. It is crucial to select a healthy plant that is in the early stages of its vegetative phase to guarantee its resilience against the stress of topping and training. Most cultivators delay until the seedling has formed approximately 5–6 nodes (25 cm), which, contingent upon nutrient and light availability, should occur within two weeks of the commencement of vegetative growth.
3. The site on the plant where HST is to be applied should be selected. Site selection is dependent on two factors: the number of shoots that exist below the excision point and the desired height.
4. A sterile razor or sharp knife is used to sever the center apex at a 45° angle, proceeding to the third node from the base, just above the two lateral buds. Eliminating the central apex results in the formation of a Y-shaped structure, wherein the two remaining nodes serve as distribution centers for energy and nutrients to the remainder of the plant.
5. Following topping, all growth beneath the third node is removed. This transmits a signal to the plant to allocate all energy and nutrient distribution to the two remaining buds on the nodes. Instead of the core axis dominating, two primary branches remain that are identical to the severed segment. The adjacent fan leaves should remain intact, as they play a critical role in photosynthesis—assimilating carbon and producing sugars that are transported to lateral buds. These photosynthates support the plant’s ongoing growth and development.
6. A period of five days should be allowed for the plant to recuperate. Subsequently, the LST (tie-down method) is applied to the two primary shoots, which are gently secured horizontally to the ground at a 90° angle. The cultivator should maintain control over the crop's uniformity. This is followed by another period of recuperation for the plants. Recovery is determined by the observation of the photosynthetic fan leaves reorienting towards the light once more.
7. Upon the observed recovery of the two primary shoots, topping may be performed once again. During each shoot, choose two nodes and sever them thereafter. The incisions must be equidistant from the manifold, as symmetry is essential for promoting uniform plant development. Topping should be continued until the cultivator is satisfied with the manifold configuration (structure). Defoliation beneath the nodes must be maintained to facilitate energy and nutrient transfer. Plants must be inspected bi-weekly to maintain a uniform canopy during the vegetative phase.
8. During the weeks spent of plant maintenance, it is crucial to supply the correct nutrients, water and sunlight to the mainlined plant. Time intervals must be considered to ensure adequate recovery from the various stressing techniques. To promote effective healing and reduce the risk of infection at injury sites, it is essential to use clean, sharp tools to make precise cuts and maintain dry conditions. Although some growers use grafting wax or tape, these practices are anecdotal and may increase pathogen risk if not adequately applied. Scientifically sound wound care emphasizes sterile techniques and allowing the wound to dry and heal naturally, reducing exposure to microbial contaminants.
9. When *Cannabis* plants transition into the flowering stage in indoor cultivation, it is essential to adjust the photoperiod from a vegetative schedule of 16 h of light and 8 h of darkness to a 12/12 light–dark cycle. This shift not only reduces the duration of light exposure but also alters the light spectrum, increasing the proportion of red light relative to blue light within the PAR range. This change in both duration and spectral quality of radiance activates the plant’s flowering response, thereby initiating bud development. Attention should be given to the main colas and any uncontrolled growth should be removed. Topping and training must stop once the plant enters flowering.
10. As plants continue to grow till harvest, individual inflorescences laddened with THC/CBD or seeds can become too heavy to stand erect and cause the cola to bend under the weight. This is when SCROG (see Table 1) is incorporated into mainlining, as it offers support to the flowering manifold. Colas are typically ready for harvest when approximately 60–70% of the trichomes have turned amber, indicating peak cannabinoid maturity. However, the precise timing of harvest varies and is contingent on other variables, including cultivar, environmental conditions, and cultivation technique. In many outdoor settings, this may occur around 6 to 8 months after planting. It is important to note that this time frame is not universally applicable (Fig 5).

### Research gaps

Despite widespread adoption of the mainlining technique among *Cannabis* cultivators, scientific validation remains conspicuously limited. Much of the existing guidance derives from anecdotal sources such as blogs and informal online forums, creating a disconnect between empirical practice and standardized agronomic methodologies. Addressing this divide requires a concerted research effort to quantify the morphological, molecular, and biochemical consequences of mainlining across diverse *Cannabis* genotypes (Da Cunha Leme Filho et al. 2025).

A critical gap lies in the absence of standardized protocols for different *Cannabis* chemovars—namely, feminized, autoflowering, and photoperiod strains. These genotypes exhibit distinct architectural traits and stress sensitivities, making the development of genotype-specific mainlining guidelines essential. Factorial experiments designed to assess phenotypic plasticity, metabolite output, and recovery time under high-stress conditions would be instrumental in improving reproducibility and scalability (Hesami et al. 2021; Dreger et al. 2023).

Molecular profiling of mainlined plants has yet to capture the stress-induced activation of stem cell niches and hormonal redistribution patterns. Technologies such as transcriptomics, proteomics, and metabolomics are underutilized in characterizing the developmental reprogramming triggered by apical decapitation, despite evidence from meristematic models in *Arabidopsis* and *Populus* indicating the feasibility of such analyses (Hu et al. 2025; Satterlee et al. 2020; Miyashima et al. 2013).

Sensor-based automation and artificial intelligence (AI) also remain absent from mainlining research. Precision tools, including RGB imaging, hyperspectral analysis, and real-time stress sensing can be paired with machine learning algorithms to optimize training intervals, monitor plant recovery, and reduce human error (Vishal et al. 2020; Chang et al. 2021; Srivastav et al. 2025). These interventions offer the potential to transform mainlining from a manual craft into a scalable and intelligent horticultural strategy.

Furthermore, autoflowering strains present a unique challenge due to their genetically programmed shift into flowering irrespective of photoperiod. Limited studies have explored whether hormonal manipulation, particularly via auxins or cytokinins, could temporarily override this flowering schedule to enable vegetative extension and response to training. The feasibility of inducing lateral branching in these cultivars without compromising yield quality remains unknown (Bevan et al. 2021; Adhikary et al. 2022).

Equally pressing is the need to evaluate how tissue culture-derived genotypes respond to mainlining. Plants generated through micropropagation may exhibit somaclonal variation or reduced symmetry, especially under stress conditions. Investigating the compatibility of mainlining with in vitro-regenerated plantlets would provide insights into whether elite clones can be trained effectively, thereby broadening the applicability of the method in commercial propagation systems (Martín and González-Benito 2024; Adamek et al. 2024; Holmes et al. 2021).

An emerging but understudied area is the combined impact of stress stacking—integrating mainlining with abiotic stressors such as light deprivation, cold exposure, and nutrient fluctuations. The developmental plasticity of *Cannabis* may be harnessed more effectively if the synergistic or antagonistic effects of these stressors are comprehensively understood through factorial trials and dynamic modelling (Tarafder et al. 2023; Pandey et al. 2015; Mason et al. 2014).

Finally, the integration of mainlining with genomic selection and precision breeding strategies remains uncharted. Novel polyploid lines, including triploid and tetraploid *Cannabis* cultivars, are being developed for enhanced metabolite profiles. However, their physiological response to high-stress training has not been evaluated. Studies exploring structural resilience, bud uniformity, and cannabinoid composition in genetically engineered lines are needed to guide cultivar selection for advanced agronomic interventions (Malabadi et al. 2024; Parsons et al. 2019).

## Conclusion

Mainlining is a technique based on the principle of apical dominance, wherein the severance of an apically dominant *Cannabis* plant results in a directional shift of hormones, sugars, and auxins from the primary apex to the axillary (lateral) sites, which then take on the role of the central buds. Wild *Cannabis* crops exhibit uniform growth patterns, which may not yield a return commensurate with the agricultural investment. Consequently, the mainlining of *Cannabis* involves the manipulation of plant architecture to optimize the distribution of auxins, cytokinins, strigolactones, energy, and nutrients, thereby enhancing bud size and increasing overall yields. Over the years, advancements in understanding the molecular mechanisms of auxins and sugars in axillary bud development have refuted outdated assumptions regarding the exclusive role of auxins in apical dominance. The current focus in this review article is to examine the synergistic function of primary and secondary metabolites in influencing the molecular environment of the *Cannabis* plant as it undergoes numerous physical and biochemical changes during cultivation. The response of *Cannabis* plants to injury and stress is a phenomenon well-understood in botanical research. Injury and stress to *Cannabis* plants enhance their latent developmental potential and provide a method for further manipulation of the canopy to achieve desired levels of plant growth, development, and yield. Upon alteration of the canopy, the vascular system is stimulated to facilitate survival and recuperation from such intense training efforts. A significant portion of the information regarding mainlining is derived from semi-reliable sources, including online blogs, amateur tutorials, and inquisitive cultivators. Thus, presenting the need for pedagogical collation of coherent information on mainlining for academic and research guidelines and techniques in cultivation. Additionally, mainlining presents a promising avenue for yield optimization in *Cannabis sativa*, but its scientific foundation must be strengthened through multidisciplinary research that combines plant physiology, molecular biology, data science, and breeding innovation.

## Data Availability

Not applicable.
